# GROUP-BASED PARTICIPATORY ARTS INTERVENTIONS: PRODUCING ART AND VALIDATION OF PERSONHOOD

**DOI:** 10.1093/geroni/igz038.716

**Published:** 2019-11-08

**Authors:** Daniel B Kaplan, Gary Glazner

**Affiliations:** 1 Adelphi University, Garden City, New York, United States; 2 The Alzheimer's Poetry Project, New York, New York, United States

## Abstract

Participatory arts-based interventions for people living with dementia involve the collaborative creation and performance of poetry, story, song, dance, and visual arts. These programs are designed to support self-expression and productive collaboration among people living with dementia while stimulating positive social interactions and feelings of empowerment and validation. This paper explores the use and potential benefits of validation in the implementation of person-centered participatory arts interventions in the context of dementia care. The authors offer a theoretical framework for understanding validation as a common intervention method and participant experience during these interventions, organized into five themes: collaboration, connection, creation, communication, and confirmation. Basic validation techniques are easily learned and can be incorporated into a broad diversity of dementia care strategies and interventions including activity programs designed to stimulate cognitive, physical, and communicative abilities. Participatory arts programs offer tools for connecting to personhood in a population for whom such connections are under constant threat of erosion. As activity programs based in creative arts help to support self-expression among participants and serve as a vehicle for generating feelings of self-efficacy, such activities are well suited to fostering the person-centered goals of dementia care programming. Clinicians and other transdisciplinary care providers are encouraged to understand, use, and teach these and other validation-focused arts interventions with persons living with dementia. These validation experiences are suggested to offer direct clinical benefits for participants as well as indirect benefits when modeled in the presence of formal care providers and family members.

